# Protocol for a 20-year follow-up after a randomized controlled trial of a Mediterranean diet in pregnancy: maternal and offspring risk factors for cardiovascular disease

**DOI:** 10.3389/fped.2023.1256815

**Published:** 2023-10-18

**Authors:** Hannibal Troensegaard, Janette Khoury, Ane C. Westerberg, Serena Tonstad, Jeanine Roeters van Lennep, Marit B. Veierød, Per Ole Iversen, Kirsten B. Holven, Kjetil Retterstøl

**Affiliations:** ^1^ Department of Nutrition, Institute of Basic Medical Sciences, University of Oslo, Oslo, Norway; ^2^ Department of Obstetrics, Division of Obstetrics and Gynaecology, Oslo University Hospital Rikshospitalet, Oslo, Norway; ^3^ School of Health Sciences, Kristiania University College, Oslo, Norway; ^4^ Department of Preventive Cardiology, Oslo University Hospital, Oslo, Norway; ^5^ Department of Internal Medicine, Erasmus MC Cardiovascular Institute, University Medical Centre Rotterdam, Rotterdam, Netherlands; ^6^ Oslo Centre for Biostatistics and Epidemiology, Department of Biostatistics, Institute of Basic Medical Sciences, University of Oslo, Oslo, Norway; ^7^ Department of Haematology, Oslo University Hospital, Oslo, Norway; ^8^ Norwegian National Advisory Unit on Familial Hypercholesterolemia, Oslo University Hospital, Oslo, Norway; ^9^ The Lipid Clinic, Department of Medicine, Oslo University Hospital, Oslo, Norway

**Keywords:** cardiovascular risk factors, offspring health, antenatal care, lifestyle intervention, Mediterranean diet, long-term follow-up, pregnancy, randomized controlled trial

## Abstract

**Background:**

An inadequate maternal diet during pregnancy can impair offspring health and may increase the risk of cardiovascular disease later in life. The purpose of the proposed study is to assess the risk factors associated with cardiovascular disease in both mothers and their offspring 20 years following their participation in a Mediterranean diet intervention trial during pregnancy.

**Methods:**

The “Cardiovascular Risk Reduction Diet In Pregnancy” (CARRDIP) study was a randomized controlled trial performed between 1999 and 2001. The participants were randomized to adhere to either a Mediterranean diet or their regular diet during pregnancy. An extensive amount of data such as diet information, ultrasound measurements, anthropometry, and biomarkers from these mothers during pregnancy and their offspring in the neonatal period were collected. The mother–offspring pairs (*n* = 269) from the CARRDIP study will be invited to participate in a clinical examination and blood sample collection. This follow-up study, conducted 20 years after the original CARRDIP study, will investigate cardiovascular risk factors in mothers and offspring. The primary outcome will be the blood pressure of the offspring. In addition, the study will explore various aspects of cardiovascular health, including metabolic and inflammatory status, clinical history, and body composition of the participants.

**Discussion:**

Previous studies investigating the effects of nutrition during pregnancy on maternal and offspring health have been either observational studies, animal studies, or randomized controlled trials with a follow-up period of less than 5 years. This project aims to study the long-term effects of dietary intervention during pregnancy on maternal and offspring cardiovascular risk markers.

**Clinical Trial Registration:**

Clinicaltrials.gov, identifier (NCT05030922).

## 1. Introduction

Cardiovascular disease (CVD) accounts for about a third of all deaths worldwide (1). Numerous studies have demonstrated that lifestyle interventions, such as smoking, physical inactivity, and dietary habits, can reduce cardiovascular risk factors. Lifestyle intervention, as primordial and primary prevention, is a cost-effective way of combatting the burden of CVD morbidity and mortality (2, 3). Although the main goals for primary and secondary prevention are well established, the targets of primordial prevention for CVD are not as clear. Nevertheless, initiating CVD prevention as early as possible, including during childhood or even *in utero*, holds significant potential (3, 4).

One of the main causes of CVD is atherosclerosis, a progressive, lifelong process resulting in plaque accumulation and inflammation within the arterial wall, which in turn may lead to thrombosis and stenosis, thereby impeding local blood flow (5). Lifestyle changes, specifically diet, influence the progression of atherosclerosis and stenosis (6, 7). For example, strong evidence demonstrates that replacing saturated fatty acids with polyunsaturated fatty acids in adults reduces the risk of coronary heart disease, as discussed in a systematic review of current evidence by the Secretary of Health and Human Services in the United States (8).

A Mediterranean diet has been shown to alter the atherosclerotic process and protect against CVD (9–12). There are several variations of the Mediterranean diet, but all are based in principle on increased intakes of whole grain bread, fruit, vegetables, legumes, and sources of polyunsaturated fat such as nuts, fish oil, and olive oil. In addition, this dietary approach involves reducing the intake of pastries, meat, and fat-rich dairy products.

The development and progression of atherosclerotic lesions are dependent on individual lifestyle choices. Notably, epigenetic factors promoting atherosclerosis may be passed on from a pregnant mother to her fetus. A pronounced maternal hypercholesterolemia during pregnancy may adversely affect both the mother and the fetus. Increased levels of cholesterol in the maternal bloodstream can accelerate the development of atherosclerotic lesions in the child during the early stages of life. Observational studies have shown that fatty streak formations in the fetal aorta are modulated by maternal hypercholesterolemia. Some observational studies have shown that signs of atherosclerosis appear in early adulthood (13–17). Furthermore, maternal cholesterol levels correlate with offspring cholesterol levels at many stages of development, including 6 months, 1 year, 2 years, and 6 years, which may increase the long-term risk of CVD (18–20).

Observational studies have found associations between maternal dietary intake during pregnancy and the body composition and cardiovascular health of the child. The composition of fatty acids in the maternal blood has been suggested to be related to childhood adiposity, and a Mediterranean dietary pattern during pregnancy has been linked to lower waist circumference in childhood (21–24). Although several randomized controlled trials (RCTs) have examined the short-term effect of dietary and lifestyle interventions in pregnancy on the health status of infants, there is a lack of long-term follow-up examining these relations in an RCT setting. According to a Cochrane review of interventions in pregnancy, a long-term follow-up is rarely done, and we were unable to identify any RCT with dietary interventions during pregnancy with a follow-up period of longer than 5 years (25).

The Cardiovascular Risk Reduction Diet in Pregnancy (CARRDIP) study was an RCT that examined the effects of a Mediterranean diet intervention on both maternal and fetal outcomes among healthy pregnant women (26–30). Some important study findings were identified in this study, including differences in the fatty acid composition in the blood and a reduction of low-density lipoprotein levels in the serum during pregnancy. In addition, the intervention group exhibited a decrease in the instances of premature births compared with the control group. While the CARRDIP study focused on the immediate health effects, we now want to examine the long-term health effects on both mothers and offspring in the CARRDIP cohort. In this study, we describe the protocol for CARRDIP20, a follow-up study conducted 20 years after the original randomized controlled trial involving dietary intervention in pregnancy.

## 2. Methods and analyses

### 2.1. Aim

**Figure 1:**
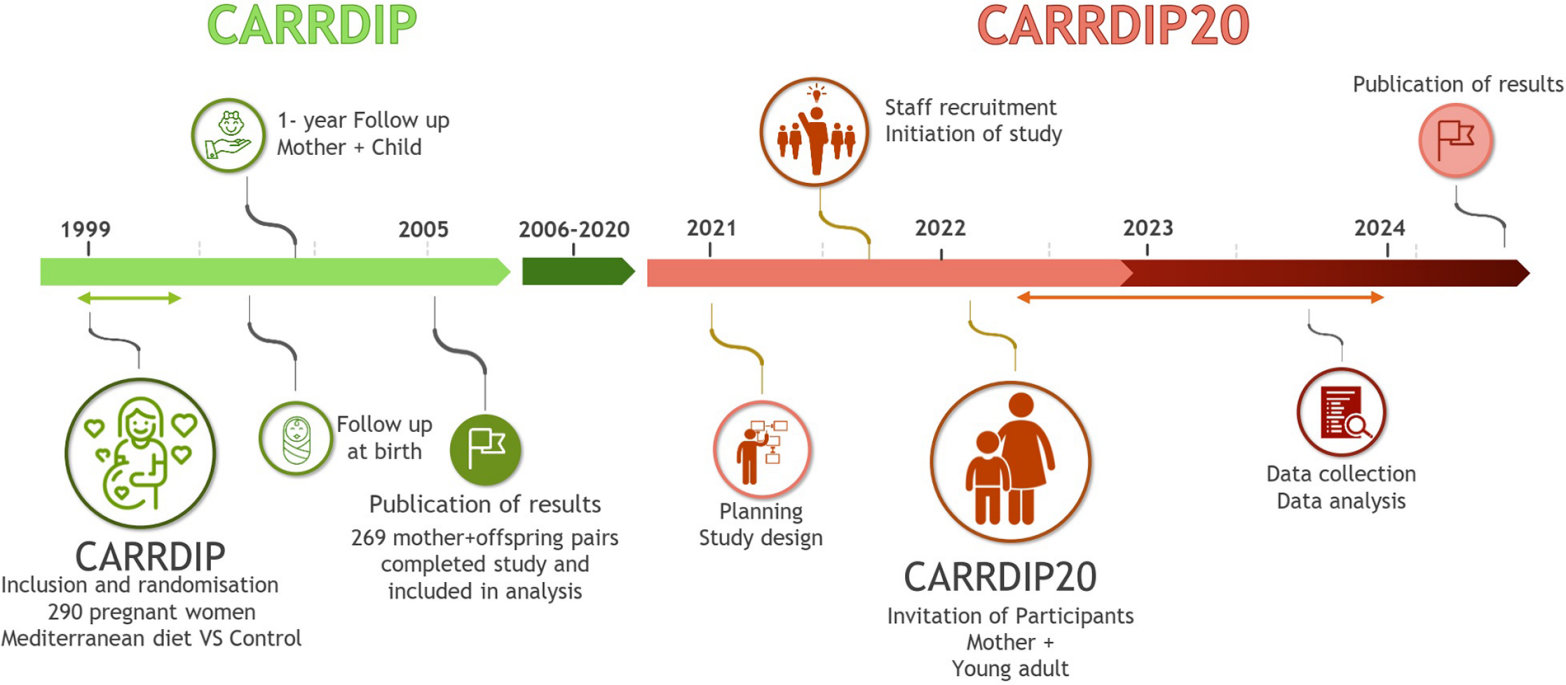
Illustration of the timeline of the original CARRDIP study and the proposed CARRDIP20 study.

This study aims to examine cardiovascular risk factors in mothers and their offspring 20 years after the mothers participated in the CARRDIP study (Figure 1) (26–28).

### 2.2. Design and setting

The CARRDIP study was an RCT with dietary intervention in pregnancy performed between 1999 and 2001. The participating mothers were allocated to either the intervention group (*n* = 141) or the control (*n* = 149) group. The aim of the intervention group was to limit dietary cholesterol to 150 mg/day and reduce the intake of saturated fat to 8% of the total energy intake. The intended composition of the total energy intake consisted of 32% from total fat, 16%–17% from protein, and 50%–51% from carbohydrates. The intervention group was encouraged to increase the intake of fatty fish, olive oil, nuts, and avocado to replace meat, butter, and fatty dairy products and to replace full-fat dairy products with skimmed or low-fat ones. The control group consumed their usual diet and did not introduce oils or low-fat meat or dairy products, aiming at 32% of energy from total fat (including 12% from saturated fat), 16%–17% of energy from protein, and 50%–51% of energy from carbohydrates. Total energy intake aimed at a weight gain of 8–14 kg during pregnancy in both study groups (26). The participants were monitored several times during pregnancy with planned follow-up visits at gestation weeks 24, 30, and 36. Mothers and offspring were followed up, both to ensure adherence to the Mediterranean diet in the intervention group and to monitor the health status of both the mother and the fetus (fetal biometric measurements and Doppler measurements). Blood lipid levels, markers of endothelial activation, and inflammation were measured in maternal samples during pregnancy and in cord blood at birth. Several pregnancy and birth outcomes were recorded, and neonatal lipid levels were measured. The CARRDIP study found a significant reduction in the umbilical artery pulsatile index, reflecting decreased placental vascular resistance (27). This reduction in the index between gestational weeks 24 and 30 was a significant predictor of higher systolic blood pressure at 6 months of age, supporting the concept of fetal origins in the development of cardiovascular risk factors (30).

The current CARRDIP20 study will be a long-term follow-up of all mothers and their offspring separately, comparing the effects of intervention against control in both groups.

### 2.3. Participants

The CARRDIP study included healthy, low-risk pregnancies to minimize possible confounding factors related to later CVD risk in the offspring. Eligible participants were included after an ultrasound showing a single healthy fetus in gestational weeks 17–20. The inclusion criteria were non-smoking pregnant women aged 21–38 years with a body mass index (BMI) of 19–32 kg/m^2^. The exclusion criteria were high-risk pregnancy due to diabetes mellitus, endocrine disease, chronic hypertension, drug abuse, history of thromboembolic disease, or significant gastrointestinal, cardiac, pulmonary, or hematologic disease. Of the 290 women included in the CARRDIP study, 21 women withdrew their consent before or during the intervention, leaving a final number of 269 mother–offspring pairs. All mothers and their offspring from the CARRDIP cohort will be invited to participate in the follow-up.

### 2.4. Data collection

The mother–offspring pairs from the original CARRDIP study will be invited to participate in a clinical examination and sample collection at the Department of Nutrition, Institute of Basic Medical Sciences, University of Oslo. Study investigators will be blinded to the original study group allocation. Data to be collected during the visit are presented in Table 1.

**Table 1:** Data to be collected in the CARRDIP20 study both from the mothers and their offspring.

Data to be collected	Analysis/methodology
Blood biomarkers	Lipid profiles (total cholesterol, non-high-density lipoprotein cholesterol, low-density lipoprotein cholesterol, triglycerides, and apolipoprotein A + B including ratio, lipoprotein(a)), markers of glucose metabolism, ferritin, liver transaminases, TSH, creatinine, tissue plasminogen activator antigen, cellular adhesion molecules, proinflammatory markers (e.g., CRP, TNFα, interleukins, interferons), hemostatic markers (e.g., fibrinogen, tissue factor, D-dimer), and differential white blood cell count
Peripheral blood mononuclear cells (PBMC) transcriptomics	PBMC will be isolated, and gene expression analysis will be performed. We will investigate both the global gene expression profile and targeted gene expression analysis, particularly investigating genes involved in lipid metabolism and inflammatory pathways
Blood pressure	Systolic and diastolic blood pressure as recorded ambulatory with appropriate devices
Dietary intake and health score	Dietary intake will be assessed with a validated food frequency questionnaire (31). Health score will be determined with the Cardiovascular Health Score of the American Heart Association (32).
Anthropometry	Weight, height, waist circumference, and hip circumference using scales and non-stretch tapes
Body composition	Fat percentage of body weight and skeletal muscle mass.
Intima-media thickness (IMT) and liver fat content	An ultrasound investigation of the IMT of the carotid artery offers a possibility to early identify the risk of atherosclerotic lesions. An ultrasound of the liver will reveal fat deposition (which is associated with several non-communicable diseases)

### 2.5. Outcomes

The primary and secondary outcomes will be based on the findings and sampling methods of the original study and of similar studies on nutritional status during pregnancy (23, 26–28, 30, 33–39). The assessment, analysis, and reporting of outcomes will follow relevant CONSORT guidelines for clinical trials, and the current study protocol will adhere to the SPIRIT (Standard Protocol Items: Recommendations for Interventional Trials) guidelines (40, 41).

The primary outcome of the study will be the difference in the mean arterial blood pressure between the control and intervention groups in the CARRDIP offspring group due to hypertension being one of the most important CVD risk factors (42). Maternal exposures during pregnancy have been found to potentially influence offspring's blood pressure and contribute to the development of CVD risk factors later in life. Factors such as maternal smoking, alcohol consumption, or diet have been associated with an increased risk of elevated blood pressure levels in the offspring. In addition, these maternal exposures have been linked to developing other CVD risk factors in the offspring, including dyslipidemia, insulin resistance, obesity, and impaired glucose metabolism (33, 43, 44). Moreover, we have previously collected blood pressure measurements among the offspring at both 1-year and 3-year follow-up visits. Thus, the blood pressure variable presents an opportunity for further analysis on parameters tracking from birth, through childhood, and into early adulthood.

The following secondary outcomes will be differences in the intervention and control groups in both mothers and offspring: lipid profile, fasting glucose and insulin, HbA1c, markers of endothelial activation and inflammation such as tissue plasminogen activator antigen (tPAag), von Willebrand factor (vWF), plasminogen activator inhibitor-1 (PAI-1) activity, plasminogen activator inhibitor-2 (PAI2) antigen, C-reactive protein (CRP), and differential white blood cell (WBC) count. We will use appropriate cardiovascular risk scores to estimate cardiovascular risk status and compare control and intervention in both the mothers and offspring separately. Additional secondary outcomes will include differences in intima-media thickness in the carotid artery and liver fat content measured by ultrasound, variations in body composition indicators by analyzing fat percentage, and skeletal muscle mass measured by bio-impedance. In addition, measurements of waist circumference, hip circumference, and body mass index will be conducted.

#### 2.5.1. Sample size

The CARRDIP randomized trial was designed to detect a clinically relevant difference in maternal cholesterol levels at week 36 in pregnancy and in neonatal cholesterol levels (26). In the CARRDIP20 study, we examined differences in CVD risk factors, which are typically in the range of 0.5–0.7 standard deviations (SDs) in intervention trials with groups randomized to different diets (45), requiring a sample size of approximately 64–100 per group with 80% power at a 5% significance level. Thus, despite the expected loss to follow-up, we most likely have statistical power to detect clinically relevant differences between the groups (46). If we assume an average difference in the systolic blood pressure of 5 mmHg, with SD = 10 mmHg, we will need 128 participants (64 in each group; power 80%, significance level 5%). This corresponds to 47% of the mothers that participated in the CARRDIP trial.

#### 2.5.2. Data analysis

The differences between the intervention and control group in primary and secondary outcomes will be analyzed separately for the mothers and offspring. We will follow the CONSORT guidelines (47–49). The primary outcome, blood pressure, and the majority of the secondary outcomes are continuous with two time points: baseline and a 20-year follow-up. Analysis of covariance will be used to analyze the data (50). There may be some selection of participants in a long-term follow-up. We can adjust for variables that differ between the intervention and control group and are associated with the outcome of interest (48). However, the use of analysis of covariance (i.e., adjustment for the baseline value) may have to be evaluated if there are major indications of selective drop-out. In that case, we will analyze the difference between the values observed at the 20-year follow-up and the baseline using a multivariable linear regression model and adjust for potential covariates.

## 3. Discussion

The existing evidence suggests that maternal diet during pregnancy may impact the health status of the offspring. Moreover, observational studies support the significance of the *in utero* metabolic environment on the risk of CVD in the offspring later in life (51, 52). The data from the randomized controlled CARRDIP trial with significant clinical effects in both mother and offspring offer a unique possibility to study possible mechanisms for the observed differences. Furthermore, it is crucial to identify possible targets for appropriate dietary intervention during pregnancy to optimize the health of the offspring, both *in utero* and later in life. Ideally, these potential targets should be investigated through long-term follow-ups of RCTs.

To date, studies investigating the effects and mechanisms of diet on the long-term health of the child have primarily been observational and animal studies. Thus, the proposed CARRDIP20 study of the long-term (20 years) effects of a Mediterranean diet during pregnancy on offspring health is unique and of potential great clinical and scientific interest.

Pregnancy may be regarded as a “window of opportunity” where interventions can promote the long-term health outcomes of both the mother and the offspring. Further identification of mechanisms responsible for fetal programming of adult CVD may identify possibilities for targeted treatment among groups at risk of CVD, as well as improved dietary recommendations and policy strategies to improve health in the general population.

## Data Availability

The datasets generated and analyzed during the current study will not be publicly available due to privacy concerns of participants in the study. Restrictions apply to the availability of these data, which were used under license for the current study, but are available from the corresponding author on reasonable request and with permission from Committees for Medical and Health Research Ethics, Norway.
